# Enhanced simultaneous adsorption of As(iii), Cd(ii), Pb(ii) and Cr(vi) ions from aqueous solution using cassava root husk-derived biochar loaded with ZnO nanoparticles

**DOI:** 10.1039/d1ra01599k

**Published:** 2021-05-25

**Authors:** P. T. Tho, Huu Tap Van, Lan Huong Nguyen, Trung Kien Hoang, Thi Ngoc Ha Tran, Thi Tuyet Nguyen, Thi Bich Hanh Nguyen, Van Quang Nguyen, Hung Le Sy, Van Nam Thai, Quoc Ba Tran, Seyed Mohsen Sadeghzadeh, Robabeh Asadpour, Phan Quang Thang

**Affiliations:** Laboratory of Magnetism and Magnetic Materials, Advanced Institute of Materials Science, Ton Duc Thang University Ho Chi Minh City Vietnam phamtruongtho@tdtu.edu.vn; Faculty of Applied Sciences, Ton Duc Thang University Ho Chi Minh City Vietnam; Faculty of Natural Resources and Environment, TNU - University of Sciences (TNUS) Tan Thinh Ward Thai Nguyen City Vietnam tapvh@tnus.edu.vn; Faculty of Environment - Natural Resources and Climate Change, Ho Chi Minh City University of Food Industry (HUFI) Ho Chi Minh City Vietnam; The Center for Technology Incubator and Startup Support, Thai Nguyen University of Agriculture and Forestry Quyet Thang Ward Thai Nguyen City Vietnam; Advanced Educational Program, Thai Nguyen University of Agriculture and Forestry Quyet Thang Ward Thai Nguyen City Vietnam; HUTECH Institute of Applied Sciences, Ho Chi Minh City University of Technology (HUTECH) 475A Dien Bien Phu, Ward 25, Binh Thanh Dist Ho Chi Minh City Vietnam; Institute of Research and Development, Duy Tan University Da Nang 550000 Vietnam tranbaquoc@duytan.edu.vn; Faculty of Environmental and Chemical Engineering, Duy Tan University Da Nang 550000 Vietnam; New Materials Technology and Processing Research Center, Department of Chemistry, Neyshabur Branch, Islamic Azad University Neyshabur Iran; Geosciences & Petroleum Engineering Department, Universiti Teknologi PETRONAS 32610 Bandar Seri Iskandar Perak Darul Ridzuan Malaysia; Institute of Environmental Technology, Vietnam Academy of Science and Technology 18 Hoang Quoc Viet Road Ha Noi City Vietnam

## Abstract

This study presents the modification of cassava root husk-derived biochar (CRHB) with ZnO nanoparticles (ZnO-NPs) for the simultaneous adsorption of As(iii), Cd(ii), Pb(ii) and Cr(vi). By conducting batch-mode experiments, it was concluded that 3% w/w was the best impregnation ratio for the modification of CRHB using ZnO-NPs, and was denoted as CRHB-ZnO3 in this study. The optimal conditions for heavy metal adsorption were obtained at a pH of 6–7, contact time of 60 min, and initial metal concentration of 80 mg L^−1^. The heavy metal adsorption capacities onto CRHB-ZnO3 showed the following tendency: Pb(ii) > Cd(ii) > As(iii) > Cr(vi). The total optimal adsorption capacity achieved in the adsorption of the 4 abovementioned metals reached 115.11 and 154.21 mg g^−1^ for CRHB and CRHB-ZnO3, respectively. For each Pb(ii), Cd(ii), As(iii), and Cr(vi) metal, the maximum adsorption capacities of CRHB-ZnO3 were 44.27, 42.05, 39.52, and 28.37 mg g^−1^, respectively, and those of CRHB were 34.47, 32.33, 26.42 and 21.89 mg g^−1^, respectively. In terms of kinetics, both the pseudo-first-order and the pseudo-second-order fit well with metal adsorption onto biochars with a high correlation coefficient of *R*^2^, while the best isothermal description followed the Langmuir model. As a result, the adsorption process of heavy metals onto biochars was chemisorption on homogeneous monolayers, which was mainly controlled by cation exchange and surface precipitation mechanisms due to enriched oxygen-containing surface groups with ZnO-NP modification of biochar. The FTIR and EDS analysis data confirmed the important role of oxygen-containing surface groups, which significantly contributed to removal of heavy metals with extremely high adsorption capacities, comparable with other studies. In conclusion, due to very high adsorption capacities for metal cations, the cassava root husk-derived biochar modified with ZnO-NPs can be applied as the alternative, inexpensive, non-toxic and highly effective adsorbent in the removal of various toxic cations.

## Abstract 2

This study presents the modification of cassava root husk-derived biochar (CRHB) with ZnO nanoparticles (ZnO-NPs) for the simultaneous adsorption of As(iii), Cd(ii), Pb(ii) and Cr(vi).

## Graphical abstract

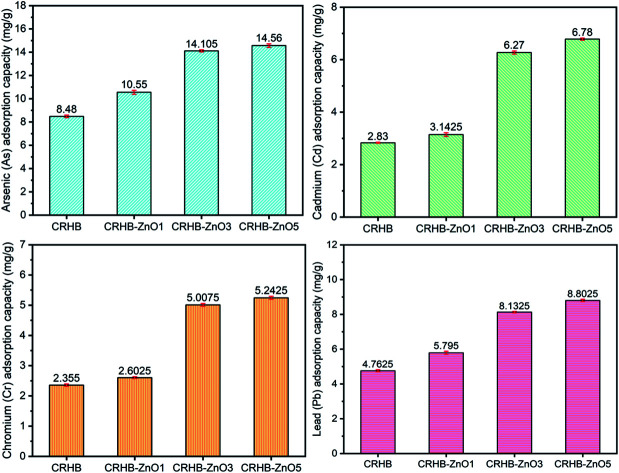

## 1. Introduction

Trivalent arsenic (As(iii)), lead (Pb), cadmium (Cd), and hexavalent chromium (Cr(vi)) are natural constituents existing in soils, groundwater, and surface water sources.^1,2^ Due to their toxicity, media containing these metals excessively are considered contaminated.^3^ In fact, aqueous environments are likely the most prevalent source of heavy metal contamination because humans directly consume water or foods containing heavy metals from water.^4^ More dramatically, industrial wastes that comprise heavy metals from exploitation and production activities are often discharged directly to streams, rivers, or oceans.^5,6^ To be more specific, for example, the metal refining industries have produced wastewater with all aforementioned metals and even other poisonous elements such as mercury (Hg).^7^ Dye production is also notorious for the potential extreme chromium pollution.^8^ Additionally, heavy metal poisoning is usually lurked in water sources that are close to mechanical industries.^6^ Regarding the effects of As(iii), Pb(ii), Cd(ii), and Cr(vi) on human being, these heavy metals are capable of damaging materials at molecular and cellular levels after penetrated into body through water or food chain.^9–12^ In other words, they cause the degeneration of vital organs and neurological disorders, facilitating related diseases. More severely, they can alter hereditary materials like DNA, causing unwanted harmful mutations to the suffering bodies and, possibly, their descendants.^13–16^ Besides, not only does heavy metal contaminated water affect humanity, it inflicts negative impacts to ecosystems as well.^10,12,17^

As a result, studies on treatments to eliminate As(iii), Pb(ii), Cd(ii), and Cr(vi) from aqueous environments should be highly considered. Until now, there have been numerous studies introducing many techniques for removing heavy metal contamination from water. Specifically, those are physico-chemical methods, such as membrane filtration, ion exchange, and adsorption.^18,19^ Membrane filtration and ion exchange have been well studied, attaining certain achievements in removing heavy metals from aqueous environments.^20–24^ However, these both techniques operate complicated and often require large amount of investment.^19,25^ On the other hand, adsorption is simpler in terms of operation and much cheaper in terms of expense.^26–28^ It also offers a significant versatility when the materials were used as adsorbents can be changed, modified, and utilized in combination. The studies of Pena *et al.*^29^ and Lou *et al.*^30^ on heavy metals adsorption onto titanium oxide and polyacrylonitrile, respectively, are remarkable for their clarification toward removing heavy metals by chemical adsorption. Whereas, Coelho *et al.* (2018)^31^ examined the modification of cashew nut shell with chemical solutions in adsorbing Cd(ii), Pb(ii), and Cr(vi) achieved promising results. Some researchers even found out that microorganisms possessed the ability to adsorb heavy metals. Particularly, in 2009, Miyatake and Hayashi^32^ published a study employing *Bacillus megaterium* to remove arsenic from aqueous solutions and successfully to determine the maximum adsorption capacity of this exotic adsorbent (0.127 mg g^−1^) and its isothermal description. The study of García *et al.*^33^ further asserted the applicability of *Bacillus* species in treating other heavy metals (Cd, Cr, and Pb).

Recently, a new trend of heavy metal adsorption has been emerged which has applied low-cost biochars derived from agricultural by-products.^34–37^ For instance, carbonaceous-rich materials from pyrolysis of chicken bones were producted to remove pollutants from wastewater.^38^ Fertilizer industry effluent was also adsorbed by carbon nanotubes stabilized in chitosan sponge.^39^ Many studies have proven the potentials of biochars for the adsorption of heavy metals in aqueous solutions. To be specific, Agbozu and Emoruwa^41^ investigated the performance of coconut husk in adsorbing different heavy metals (Cd(ii), Cr(vi), Pb(ii), *etc.*) and obtained positive adsorption capacities. While Alam *et al.*^42^ and Sarmah *et al.*^43^ declared successfully in application of golden shower (*Cassia fistula*)-derived biochar and paddy husk ash, respectively, in the removal of As(iii) and As(v) by adsorption. There was even a study that employed rice husk as an adsorbent for removal of heavy metal.^40^ These are solid fundaments to implement a study that focuses on another agricultural by-product, cassava root husk, which was considered only as a waste in agricultural production process.

However, the universally low adsorption capacity of agricultural residuals-derived adsorbents was proved which caused the use of an excessive amount of materials for practically removing contaminants. Zinc oxide nanoparticles (ZnO-NPs) were found to be one of the most effective materials for the modification of agricultural wastes used for adsorption mainly because this material is affordable and manufactured widely due to many applications in different fields.^44^ Regarding adsorption, ZnO nanoparticles have been employed successfully in the removal of dyes^45–47^ thanks to their large surface area and a high porosity with small particle size.^48^ Moreover, it has been reported that ZnO-NPs modification adsorption materials have possessed hydroxyl functional groups that can effectively adsorbed heavy metals.^49^ Nevertheless, the ZnO modification adsorbents with aims to reduce the cost has only been studied to remove only one type of heavy metal from solution so far.^50,51^ Therefore, the combination of this nanomaterial with an agricultural waste-derived adsorbent into a nano-biochar composite for simultaneously removal of various heavy metals from wastewater is a novelty and feasible study direction.

Vietnam, particularly the North of Vietnam, is a bustling market of cassava and the abundance of cassava husk is completely valueless. Therefore, the research group intended to utilize these waste material sources to produce a novel adsorption material for removal of heavy metals ions from aqueous environment. More specifically, the waste cassava root husk (CRH) was used to produce biochar and the biochar was then modified with ZnO nanoparticles which have popularly applied for adsorption of a wide range of contaminants.^52–54^ Clearly, this is a new study idea about a composite adsorbent that has never been applied for the adsorption of a mixture of heavy metals from water. Based on knowledge obtained from literature studies, this study aims to four primary specific targets: (1) fabricating the biochar adsorbent from wasted cassava root husk (CRH) by pyrolysis process; (2) modifying the original adsorbent by loading ZnO nanoparticles on CRH-derived biochar; (3) evaluating the environmental parameters that affect the adsorption of heavy metals in aqueous solution; and (4) simulating the adsorption behaviors of both pristine and modified biochars through typical adsorption isothermal and kinetic models. Specially, the mechanisms of heavy metals adsorption onto CRH-based adsorbents were deeply discussed in this study.

## 2. Materials and methods

### 2.1. Materials

Cadmium nitrate tetrahydrate (Cd(NO_3_)_2_·4H_2_O), K_2_Cr_2_O_7_ were purchased from Merck company (Germany) while Pb(NO_3_)_2_ and sodium arsenite (NaAsO_2_·7H_2_O) were obtained from Sigma-Aldrich (USA) and Sinopharm Chemical Reagent Co., Ltd., Shanghai, China, respectively. All the chemicals were analytical grade and were used as received without further purification. Four types of heavy metal ions, including As(iii), Cd(ii), Pb(ii), and Cr(vi) were prepared by dissolving appropriate amounts of corresponding chemical compounds in deionized water. The solutions of NaOH 0.1 M and HCl 0.1 M (Merck, Germany) were used as regulators of pH value. Pure zinc (Zn) rods and zinc oxide (ZnO) were obtained from Sigma-Aldrich (USA). Raw cassava root husks were collected from small private production facilities in Thai Nguyen Province, Vietnam.

#### Preparation of biochars

At the beginning of the fabrication process of biochar, the raw materials (*i.e.* cassava root husk – CRH) were cleaned with water and subsequently dried at temperature of 105 °C for 48 hours to stable dry weight. The obtained dried CRH was then ground to achieve a smaller size of 1–2 cm per piece. To generate cassava root husk-derived biochar (CRHB), a furnace (Nabertherm, model L3/11/B170, Germany) was used to perform a slow pyrolysis process over the ground husk. The temperature was set at 400 °C with a heating rate of 10 °C over a two-hour pyrolysis period. The obtained result solid part of this step was CRHB with non-homogeneous particles sizes. Thus, the biochar was continuously sieved to obtain particles size < 0.5 mm before preservation or further utilization.

#### Synthesis of ZnO nanoparticles

The preparation of ZnO nanoparticles (ZnO-NPs) was based on an electrochemical method. Specifically, the synthesis system of ZnO-NPs comprised of the zinc electrodes with purity>90% utilizing potassium chloride solution (0.5 M) as the electrolyte. The system was operated at temperature condition from 30 °C to 50 °C in a water bath with an applied voltage of 10 V regulated by a direct-current (DC) power generator (model TES-6220). After initiating the redox reaction that generated the ZnO-NPs, the system was agitated at 400 rpm by a corning PC-420D magnetic agitator. After 60 reaction min, a milky-white suspension of ZnO nanoparticles was obtained and cooled down to the room temperature (25 ± 2 °C). In the next step, the cooled suspension was continuously filtered by a polyvinylidene difluoride (PVDF) membrane with a pore size of 0.2 μm to acquire the desired size of particles. Finally, the filtered suspension was dried at 80 °C for 12 hours to eliminate excessive parts to achieve ZnO nanoparticles.

#### Preparation of composite biochar loaded ZnO nanoparticles

To combine ZnO-NPs into CRHB, the incipient wetness impregnation method was employed. The suitable amounts of ZnO-NPs were scaled before put into thermal-resistant 250 mL Erlenmeyer flasks containing 40 mL of ethanol solution. Afterward, the flasks were sonicated for 30 min. The CRHB were then added in accordance to the weight ratios between ZnO and biochar (1%, 3%, and 5%). Subsequently, the flasks were sealed for agitation for 2 hours at 80 °C using magnetic stirrer (VELP, SN: F20500162, Italy). The obtained result suspension was filtered before rinsed with distilled water until constant pH value. Finally, to obtain biochar fully loaded with ZnO-NPs, the filtered suspension was dried at 105 °C in 2 h. Corresponding to the pre-determined ratios, there were four types of adsorbents denoted as CRHB (0% ZnO-NPs), CRHB-ZnO1 (1% ZnO-NPs), CRHB-ZnO3 (3% ZnO-NPs), and CRHB-ZnO5 (5% ZnO-NPs).

#### Characterization of adsorbent

The characterization of CRH-based biochars included BET surface area, total pore volume, and functional groups or radicals available on the surface of the adsorbents. Also, changes regarding functional groups and surface morphology during adsorption were apprehended. This study employed the Brunauer–Emmett–Teller (BET) measure to qualify and quantify the surface area and the total pore volume of CRHB and CRHB-ZnO. For identifying functional groups and changes of surface functional groups, a FTIR spectrometer operating at 4000–500 cm^−1^ was used to process the Fourier Transform Infrared Spectra obtained from the adsorbent. Finally, the appraisals of surface morphology were achieved by the energy dispersive X-ray spectroscopy technique. Specifically, the X-ray spectrometer (Hitachi S-4800) recorded the data of Scanning Electron Microscope (SEM), EDX and mapping. The crystalline structure of ZnO nanoparticles and CRHB-ZnO3 was examined by X-ray diffraction pattern using XRD-D8 ADVANCE, with the Cu Kα radiation (*λ* = 1.5417 Å). The surface morphology of ZnO nanoparticles was also analyzed using a commercial FESEM instrument from S-4800 (model: HI-9039-0006).

In addition, the pH value at the point of zero charge (pH_PZC_) of the CRHB was declared as a characteristic feature using the shift method.^55^ This was an indicator for the charge in CRHB surface.

### 2.2. Batch adsorption experiments

The effect of impregnation ratios between ZnO-NPs and biochar, contact time, solution pH, and initial heavy metals concentration on the adsorption capacities of adsorbents toward each heavy metal was evaluated through a series of batch-mode experiments. The adsorption experiment was conducted in 50 mL Erlenmeyer flasks containing 0.01 g of each adsorbent type and 25 mL of each heavy metal-containing solution (As(iii), Cd(ii), Pb(ii), Cr(vi)) with the concentration depending on the design and the purpose of each experiment. An agitator (model PH-4A, China) was used to initiate the adsorption process at 120 rpm under the room temperature condition (25 ± 2 °C).

For the determination of the most suitable ZnO-NPs impregnation ratio, all four types of adsorbents (CRHB, CRHB-ZnO1, CRHB-ZnO3, and CRHB-ZnO5) were examined with solution pH of 6.28. The total concentration of four heavy metals (As(iii), Cd(ii), Pb(ii), and Cr(vi)) was maintained at 40 mg L^−1^ (10 mg L^−1^ of each heavy metal) in solution. After 60 min of adsorption time, the solution containing heavy metals was withdrawn to determine left heavy metals concentration in the filtrates using Inductively Coupled Plasma-Optical Emission Spectrometry (ICP-OES, Model: ULTIMA EXPERT, Horiba, France).

For determining the effect of various solution pH values, contact time, and initial metals concentrations, the experiments were designed with 2 employed adsorbents which were CRHB and the adsorbent selected from the previous experiment at optimal impregnation ratio. The pH values were adjusted using HCl 0.1 M and NaOH 0.1 M. The examination ranges were from 2 to 10 (standard deviation *σ* = 1) for pH, 0–180 min (*σ* = 30 from the 30^th^ min) for contact time, and 20–100 mg L^−1^ (*σ* = 10) for initial metals concentration. For each type of determination, other experimental conditions were maintained at a homogeneous point. The samples were taken out at interval time to determine left heavy metals concentration in solution after filtered by filter membrane with pore size of 0.45 μm. All experiments were conducted in triplicate.

The adsorption capacities of each heavy metal onto adsorbents were calculated by the equations below:1
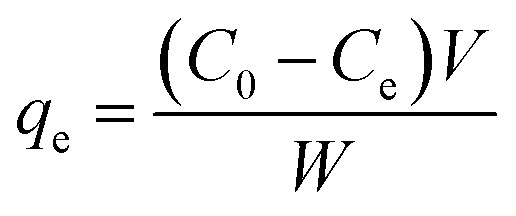
2
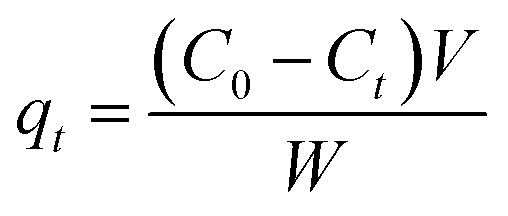
where, *q*_*t*_ is denoted the adsorption capacity at any time *t* (mg g^−1^) and *q*_e_ is for equilibrium (mg g^−1^); *C*_0_, *C*_*t*_ and *C*_e_ (mg L^−1^) are concentrations of each heavy metal at beginning time, any time *t*, and equilibrium, respectively; *W* (g) is the dry weight of CRHB or CRHB-ZnO absorbent and *V* (L) represents the volume of solution.

### 2.3. Adsorption isothermal and kinetic models

The isotherm of CRH-based adsorbents towards heavy metals was evaluated through comparison between two isothermal models, which included Langmuir and Freundlich models. To be specific, the Langmuir's isotherm states that the adsorption process occurs on only one layer of surface (monolayer) and the active sites are homogeneous in terms of energy.^56^ On the other hand, Freundlich's indicates that the energy varies on different active sites and the adsorption process is on multilayers.^57^ The expressions of these models are as the following equations:

Langmuir model: 3
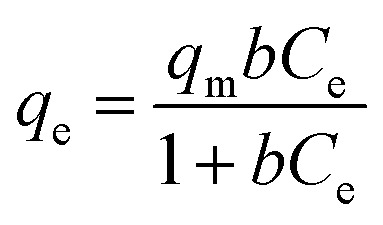


Freundlich model: 4
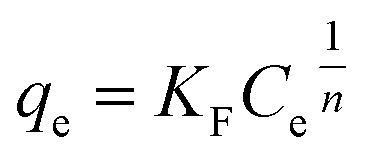
where *q*_e_ (mg g^−1^) and *q*_m_ (mg g^−1^) are the equilibrium and maximum adsorption capacities. *C*_e_ (mg L^−1^) is the concentration of the adsorbed subject at equilibrium; *b* (L mg^−1^) is the Langmuir constant, indicating the energy of the adsorption; *K*_F_ (mg g^−1^) (mg L^−1^)^*n*^ is the Freundlich constant; and *n* is the heterogeneous factor.

For the kinetics study of As(iii), Cd(ii), Pb(ii), and Cr(vi) adsorption processes, this study utilized the pseudo-first-order and pseudo-second-order models as the bases for kinetic analysis. These two models are expressed by equations as follows:

Pseudo-first-order:

5 *q*_*t*_ = *q*_e_(1 − e^−*k*_1_*t*^)

Pseudo-second-order:6
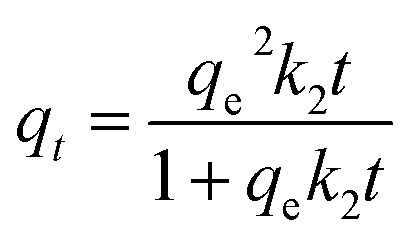
where, *q*_e_ (mg g^−1^), *q*_*t*_ (mg g^−1^), are the adsorption capacities at equilibrium and at time *t*, respectively; *k*_1_ (min^−1^), *k*_2_ (g mg^−1^ min^−1^) are the first-order rate constant and the second-order rate constant, respectively.

## 3. Results and discussion

### 3.1. The effect of impregnation ratios on heavy metal adsorption

**Fig. 1:**
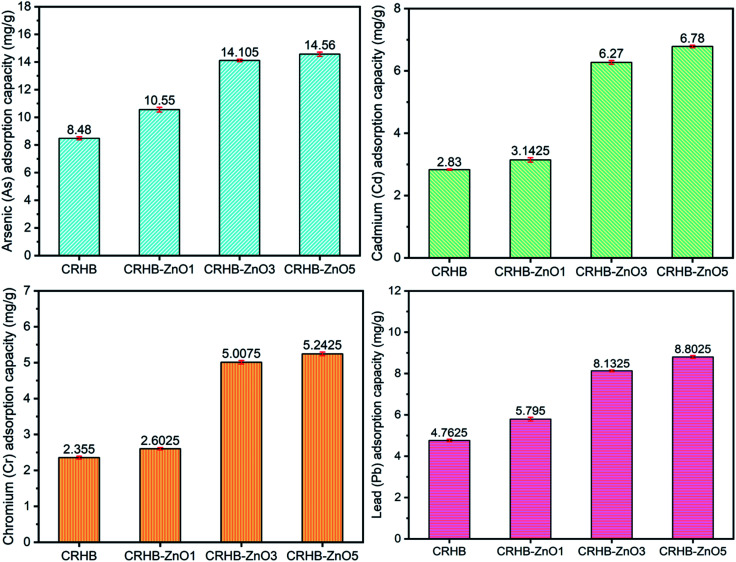
Effect of various impregnation ratios between ZnO-NPs and CRHB onto heavy metal adsorption.

This study examined four types of CRH-based adsorbents, corresponded with four impregnation ratios applied on the loading of ZnO-NPs onto cassava root husk-derived biochar. Particularly, they were CRHB (0% ZnO-NPs loaded), CRHB-ZnO1 (1% w/w ZnO-NPs loaded), CRHB-ZnO3 (3% w/w ZnO-NPs loaded), and CRHB-ZnO5 (5% w/w ZnO-NPs loaded). Adsorption experiments were conducted with each adsorbent for the mixture of total four heavy metals in solution (As(iii), Cd(ii), Pb(ii), and Cr(vi)). The experimental conditions included total initial concentrations (Pb(ii), Cd(ii), As(iii) and Cr(vi)) of 40 mg L^−1^ (the concentration of each particular metal was 10 mg L^−1^), initial pH of 6.28, contact time of 60 min, and 0.01 g of adsorbent per 25 mL solution. The adsorption capacities obtained from the performance of the employed adsorbents are presented in Fig. 1.

The increasing tendency in terms of adsorption capacity corresponding to the increase of ZnO impregnation ratios was noticeable. For all four heavy metals, pristine CRHB adsorbed the least amount while CRHB-ZnO3 outperformed all other adsorbents. At the ratios from 0% to 5%, the adsorption capacities rose from 8.48–14.56 mg g^−1^, 2.83–6.78 mg g^−1^, 4.76–8.80, and 2.35–5.24 mg g^−1^ for As(iii), Cd(ii), Pb(ii), and Cr(vi), respectively. However, determination of the most suitable impregnation ratio for loading ZnO-NPs on CRHB should be based on rate of adsorption increase among adsorbents. The results from Fig. 1 clearly indicate that the adsorption rate peaked as the ZnO-NPs impregnation ratio went to 3%. The adsorption capacity virtually remained as the impregnation ratio was 5% for adsorbing all 4 heavy metals. At the loading ratio of 3%, the adsorption capacity for As(iii), Cd(ii), Pb(ii), and Cr(vi) achieved, respectively, 14.11 mg g^−1^, 6.27 mg g^−1^, 8.13 mg g^−1^ and 5.01 mg g^−1^. Therefore, it was concluded that CRHB-ZnO3 showed the best potential for the adsorption of As(iii), Cd(ii), Pb(ii), and Cr(vi). This can be because of the amount of ZnO nanoparticles loaded on CRHB that provided more active sites on the surface of CRHB triggering the higher adsorption capacity. However, overly high impregnation ratios resulted in the growth of saturation in terms of active sites. Consequently, the adsorption capacity of the adsorbent was less effective. This tendency of the interaction between impregnation ratios and adsorption efficiency has been recorded similarly in previous studies. For instance, Hoang *et al.*^58^ demonstrated the modification of snail shell with iron nanoparticles for the adsorption of chromium(iv) in solutions. The adsorbent provided the maximum adsorption capacity at a Fe impregnation ratio of 25% and displayed no changes at higher impregnation ratios. The optimum impregnation ratio between AgNPs and activated carbon of 2% (w/w) was also determined for Cr(vi) adsorption and 0.5% for removal of methylene blue by adsorption.^59^ In conclusion, 3% was the most suitable ZnO-NPs impregnation ratio and CRHB-ZnO3 would subsequently be used in upcoming experiments.

### 3.2. The effect of pH on heavy metal adsorption

**Fig. 2:**
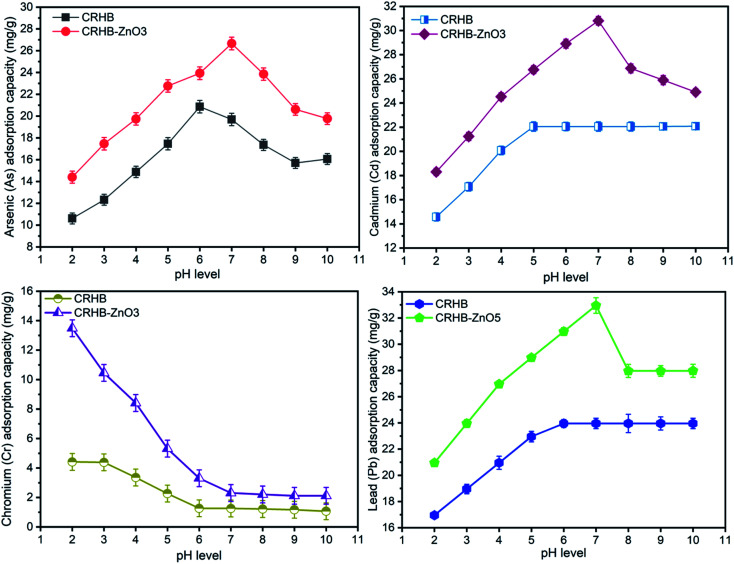
Effect of initial solution pH on heavy metals adsorption by CRHB and CRHB-ZnO3.

As described, the range of pH for examination was from 2–10 with recording on each one level. The investigated biochars for adsorption of As(iii), Cd(ii), Pb(ii), and Cr(vi) were CRHB and CRHB-ZnO3 concluded from the previous experiment. Other experimental conditions were maintained with 40 mg L^−1^ of four heavy metal concentrations, contact time of 60 min, and an adsorbent dose of 0.01 g per 25 mL solution. Recorded adsorption capacities are exhibited in Fig. 2.

The adsorption of four heavy metals with CRH-based adsorbents saw a distinct trend corresponding with different pH values as illustrated in Fig. 2. In general, the adsorption of As(iii) recorded a significant growth with a increase in pH values from 2 to 6 for CRHB and from 2–7 for CRHB-ZnO3. Specifically, the adsorption performance of CRHB grew from 10.62 mg g^−1^ (pH 2) to the peak at 20.87 mg g^−1^ (pH 6) and that of CRHB-ZnO3 was from 14.41 mg g^−1^ (pH 2) to peak at 26.67 mg g^−1^ (pH 7). Nonetheless, at higher pH levels (pH 7–9) resulted in a sharp decrease in As(iii) adsorption capacities and the adsorption capacity of As(iii) onto biochar was unchanged at higher pH values (pH of 10). For the adsorption of Cd(ii), the adsorption capacities rocketed when pH rose from 2–5 for CRHB and from 2–7 for CRHB-ZnO3. The capacity of CRHB increased from 14.58 to 22.06 mg g^−1^ (pH 5), while the capacity of CRHB-ZnO3 grew from 18.31–30.81 mg g^−1^ at pH equal 7. At higher pH levels, the capacity of CRHB remained unchanged while the adsorption performed by CRHB-ZnO3 significantly decreased. Therefore, it can be concluded that pH 7 was the peak of CRH-based adsorbents for the adsorption of Cd(ii). The interaction between Pb(ii) and CRH-based adsorbents particles with the increase of pH values relatively resembled the fluctuation tendency of the adsorption of Cd(ii). At pH from 2–7, adsorption capacities of CRHB and CRHB-ZnO3 for Pb sharply rose from 16.96–23.96 mg g^−1^ and 20.96–32.96 mg g^−1^, respectively. The increase of CRHB's adsorption capacity virtually stopped afterward and the adsorption efficiency was kept constantly. While the adsorption performance of CRHB-ZnO3 considerably dropped at pH higher than 6. Consequently, pH of 7 also the optimal point of Pb(ii) adsorption. On the contrary to adsorption tendency of other metals, the adsorption of Cr(vi) onto biochars had a completely different trend. Specifically, when pH levels rose from 2–9, the recorded adsorption capacities of both CRHB and CRHB-ZnO3 fall from 4.42–1.07 mg g^−1^ and 13.49–2.12 mg g^−1^, respectively. The Cr(vi) adsorption efficiency peaked in acidic medium (pH of 2).

Summarily, from results presented in Fig. 2, it can be seen that CRH-based adsorbents modified by ZnO-NPs exhibited the most excellently behavior for the adsorption of Cd(ii) and Pb(ii). The maximum adsorption capacities of the employed adsorbents for adsorbing these two metals achieved, respectively, 30.81 mg_Cd_ g^−1^ and 30.97 mg_Pb_ g^−1^. Besides, the different interaction tendencies of the four metals towards the used adsorbents occurred due to the difference in existing states of heavy metals ions in aqueous environment, which were mostly positive ions (As(iii), Cd(ii) and Pb(ii)) causing a low adsorption efficiency in acidic medium. The result can be explained that at low pH levels, hydrogen ions (H^+^) exhibited a strong competition with heavy metals cations on the active sites of adsorbents which resulted in a drop in adsorption capacities.^44^ Whereas, at neutral or slightly alkaline pH led to a better adsorption or unchanged adsorption because there was no competition occurring between absorbates particles and absorbents. At high pH levels, on the other hand, the charge of the adsorbent's surface was altered following tendency benefited for metals cations adsorption by negative charged surfaces of adsorbents. As the results, the adsorption capacity increased.

Moreover, the recorded pH_PZC_ values of both CRHB and CRHB-ZnO3 were, respectively, 8.25 and 6.94 which were higher than those of solution pH values proved that the surfaces of CRHB and CRHB-ZnO3 were negatively charged which favored the adsorption of cations. At acidic condition, the competition between H^+^ ions and heavy metals cations was escalated leading to lower adsorption capacity. While at alkaline condition, the As(iii), Cd(ii) and Pb(ii) cations were easily formed precipitates with OH^−^ as As(OH)^2+^, Cd(OH)^+^, and Pb(OH)^+^.^60–62^ In this study, the adsorption capacity of employed adsorbents was optimized at pH of 7, suggesting feasibility in practical application for metals removal from wastewater. More specifically, at that range, the charge of adsorbents is neutral-negative proved a strong affinity towards the metal cations. The analogue results were also reported in other studies.^63,64^ Thus, the range of pH from 6–7 was best for the adsorption of As(iii), Cd(ii), and Pb(ii) in this work.

On the other hand, the hexavalent chromium adsorption onto CRH-based biochars possessed a completely different trend. The literature references showed the existing states of Cr(vi) element strongly depended on solution pH. Specifically, Cr(vi) often exists in forms of anions HCrO_4_^−^, CrO_4_^2−^ and Cr_2_O_7_^2−^. At the range pH of 2.0–6.0, the free adsorption energy of HCrO_4_^−^ is within 2.5–0.6 kcal mol^−1^, which is lower than that of CrO_4_^2−^, which is within 2.1–0.3 kcal mol^−1^. As a result, at the same concentration, HCrO_4_^−^ is adsorbed more easily than CrO_4_^2−^. In addition, the reduction of Cr(vi) to Cr(iii) results in a better adsorption than that of Cr(vi) thanks to precipitation with –OH groups onto biochars' surface. Moreover, at solution pH levels lower than pH_PZC_, the surface of adsorbents tends to adsorb anions. As ionic forms of chromium in water are anions, the electrostatic force and linkages of anions Cr(vi) to acidic functional groups are dominant leading to enhancement of its affinity toward the adsorbents particles at the low pH levels. As a result, the adsorption capacity to hexavalent chromium was higher at lower pH.

The adsorption trend of Cd(ii), As(iii), and Pb(ii) relatively resembled the study of Agbozu and Emoruwa (2014),^41^ who examined the performance of coconut husk in adsorbing various heavy metals. Whereas the increase trend of Cr(vi) adsorption at low pH values was also observed analogously in the study of regarding Cr(vi) adsorption by coconut shell charcoal and commercial activated carbon^65^ and porous zinc-biochar nanocomposites.^50^ The optimal range from 6–7 was similar to the range of pH from 5–7 concluded by Horsfall Jr and Spiff,^66^ who studied the effects of pH on Pb(ii) and Cd(ii) sorption performed by caladium bicolor biomass. Other studies showed the similar tendencies in adsorbing heavy metals in aqueous environments. Remarkably, the optimal pH was determined to be 6 for the adsorption of cadmium and lead and 2 for the adsorption of hexavalent chromium with rice husk as the adsorbent.^40^ As the conclusion for the effect of pH on the simultaneous adsorption of four heavy metals onto CRH-based biochars, the optimal interval of pH was 5–7 for As(iii), Pb(ii), and Cd(ii) while optimal pH for adsorbing Cr(vi) was at low levels. In order to synchronize the experimental conditions, pH level 6.0 was selected as the optimal pH for subsequent adsorption experiments of heavy metals from aqueous solution in the next experiments.

### 3.3. The effect of contact time and adsorption kinetic studies

**Fig. 3:**
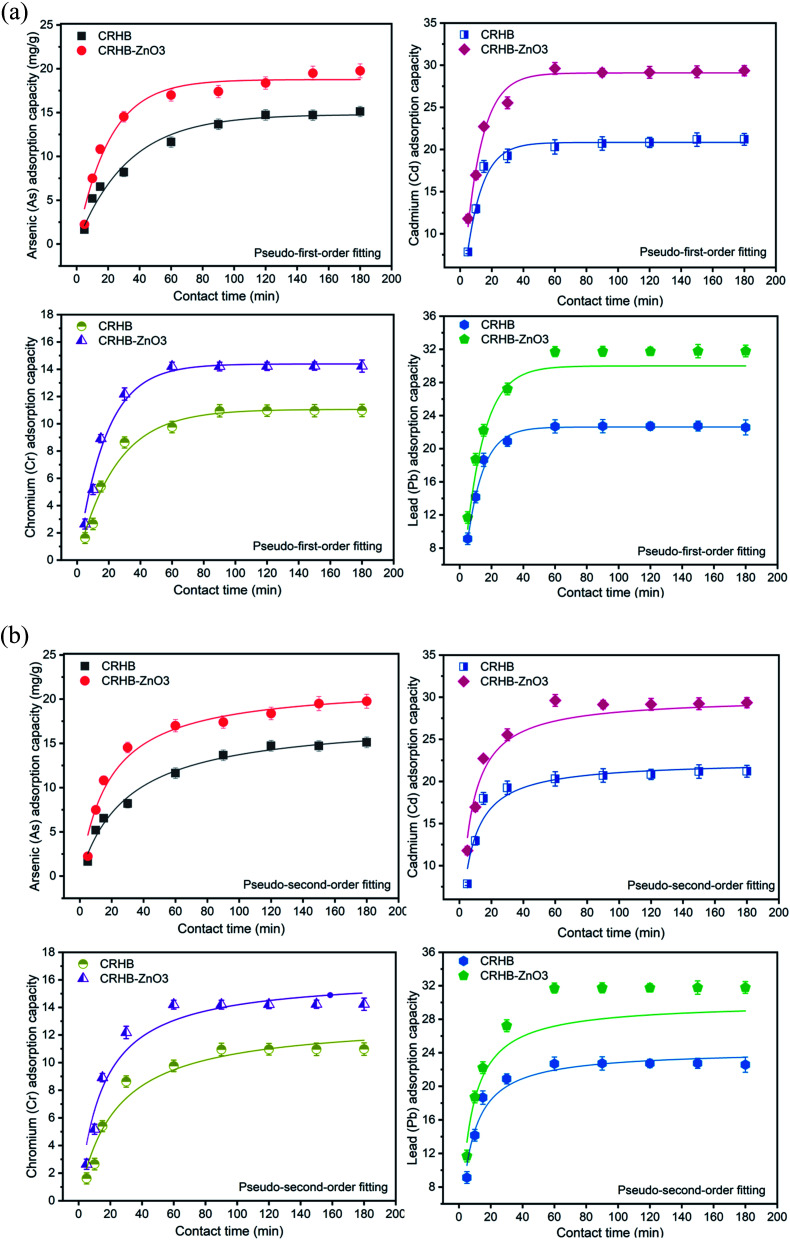
(a) Pseudo-first-order model of As(iii), Cd(ii), Pb(ii), and Cr(vi) adsorption onto CRHB and CRHB-ZnO3. (b) Pseudo-second-order model of As(iii), Cd(ii), Pb(ii), and Cr(vi) adsorption onto CRHB and CRHB-ZnO3.

The experiments to examine effect of contact time on the metal adsorption were conducted with varying of contact time from 0–180 min in order to determine the optimal contact time for the simultaneous adsorption of As(iii), Cd(ii), Pb(ii), and Cr(vi) onto CRHB and CRHB-ZnO3. Experimental flasks were maintained in terms of experimental conditions, which included pH of 6, an initial heavy metals concentration of 40 mg L^−1^ (the concentration of each metal is 10 mg L^−1^), and an adsorbent dose of 0.01 g per 25 mL solution. The adsorption capacities of metals onto biochars during contact time of 180 min are expressed in Fig. 3a.

For both adsorbents (CRHB and CRHB-ZnO3), the adsorption capacities of all adsorption processes shared a relatively analogous tendency. To be specific, in the first 30 min, adsorption substantially accelerated. In the next 30 min (the 30^th^ min to the 60^th^ min), the growth rate was slower but still was significant. However, from after the 60^th^ min reaction, recorded adsorption capacities were maintained unchanged or even slightly decreased. Explaining for this tendency, it was due to the availability of active sites on adsorbents that shifted through reaction time. In the beginning (first 30 min), there was an abundance of active sites. That was why heavy metals quickly occupied the adsorbent and the adsorption capacity increased as a result.^67^ As the availability narrowed down, the rate of acceleration dropped until all active sites were saturated at the 60^th^ min and the growth completely stopped at 80 min of reaction time.^68^ In this experiment, although all the peaks were observed at the 150^th^ to 180^th^ min but their rates of acceleration from the 60^th^ min were extremely inconsiderable. This was why the optimal time for CRH-based adsorbent to adsorb As(iii), Cd(ii), Pb(ii), and Cr(vi) should be 60 min. At the 60^th^ min, both the adsorbents displayed an order of adsorption affinity of Pb(ii) > Cd(ii) > As(iii) > Cr(vi). Specifically, the adsorption capacity of CRHB for As(iii), Cd(ii), Pb(ii), and Cr(vi), respectively, reached 11.64 mg g^−1^, 20.31 mg g^−1^, 22.70 mg g^−1^, and 9.76 mg g^−1^. While for CRHB-ZnO3, these values were 17.41 mg g^−1^, 29.62 mg g^−1^, 31.73 mg g^−1^, 14.20 mg g^−1^, respectively. This conclusion was relatively similar in comparison to the study of Alam *et al.*^42^ who concluded an optimal time of 50 min for As(iii) adsorption by golden shower biochar, and the study of Kołodyńska *et al.*^69^ who shared the same trend of optimal reaction time for adsorption of Cu(ii), Zn(ii), Cd(ii) and Pb(ii) onto pig and cow manure biochar.

The kinetics of heavy metals adsorption onto CRH-based biochars were examined by fitting experimental data with the pseudo-first-order (PFO) and the pseudo-second-order (PSO) kinetic models and their compatibility with the results obtained from the contact time experiments are illustrated in Fig. 3a and b. The kinetics calculated parameters are displayed in Table 1.

**Table 1:** Calculated kinetic parameters of heavy metals adsorption onto CRHB and CRHB-ZnO3

	As(iii)		Cd(ii)		Pb(ii)		Cr(vi)	
	CRHB	CRHB-ZnO3	CRHB	CRHB-ZnO3	CRHB	CRHB-ZnO3	CRHB	CRHB-ZnO3
**Pseudo first order**								
*q*_e_ (mg g^−1^)	15.56	19.63	20.94	29.11	22.63	31.61	11.04	14.41
*k*_1_ (g mg^−1^ min^−1^)	0.025	0.032	0.096	0.096	0.102	0.081	0.041	0.052
*R* ^2^	0.876	0.942	0.993	0.986	0.993	0.989	0.979	0.984

**Pseudo second order**								
*q*_e_ (mg g^−1^)	16.53	22.56	23.01	31.47	24.28	34.39	13.15	16.59
*k*_2_ (g mg^−1^ min^−1^)	0.001	0.001	0.005	0.004	0.006	0.004	0.003	0.004
*R* ^2^	0.864	0.928	0.971	0.971	0.963	0.986	0.958	0.957

The calculated adsorption capacities of the four heavy metal ions (*q*_e_) of both kinetic models (Table 1) were relatively well fitted the practical data (*q*_m,exp_ – Table 2). Specifically, the values of correlation coefficients *R*^2^ resulted from the fit models of all adsorption processes were virtually higher than 0.92 (Table 1) except the *R*^2^ values of both models for As(iii) adsorption onto CRHB (0.8759 and 0.8637, respectively). The difference among those values was also inconsiderable. Moreover, fit values of adsorption capacities were quite close to the actual adsorption capacities obtained from experimental data. The maximum adsorption capacities of CRHB and CRHB-ZnO3 were calculated from the PFO and PSO are expressed in Table 2.

**Table 2:** The maximum adsorption capacities of CRHB and CRHB-ZnO3 calculated from experimental data of contact time experiments

	Adsorption capacity (mg g^−1^)			
	As(iii)	Cd(ii)	Pb(ii)	Cr(vi)
CRHB	11.64	20.31	22.70	9.76
CRHB-ZnO3	17.41	29.62	31.73	14.20

In comparison with the maximum values of adsorption capacities obtained from the experimental data of investigation of effects of the contact time in this study (Table 2), it can be seen that the difference was insignificant. The adsorption affinity remained Pb(ii) > Cd(ii) > As(iii) > Cr(vi) in both models. The adsorption capacity calculated from the PFO was slightly closer to the practical capacity which was compared with the data of PSO although both models fitted very well in describing the adsorption of this study. Therefore, the kinetics of CRH-based adsorbents in heavy metal removal were well described by both pseudo-first-order and pseudo-second-order models. The fact that both models were quite compatible, indicating that the heavy metals adsorption mechanism onto CRH-based adsorbents was primarily based on chemisorption by interaction among involved components such as ion exchange^70^ and surface precipitation.^69^ This tendency of kinetics was also reported from other studies.^40,42,71^

### 3.4. The effect of initial concentrations and adsorption isothermal studies

**Fig. 4:**
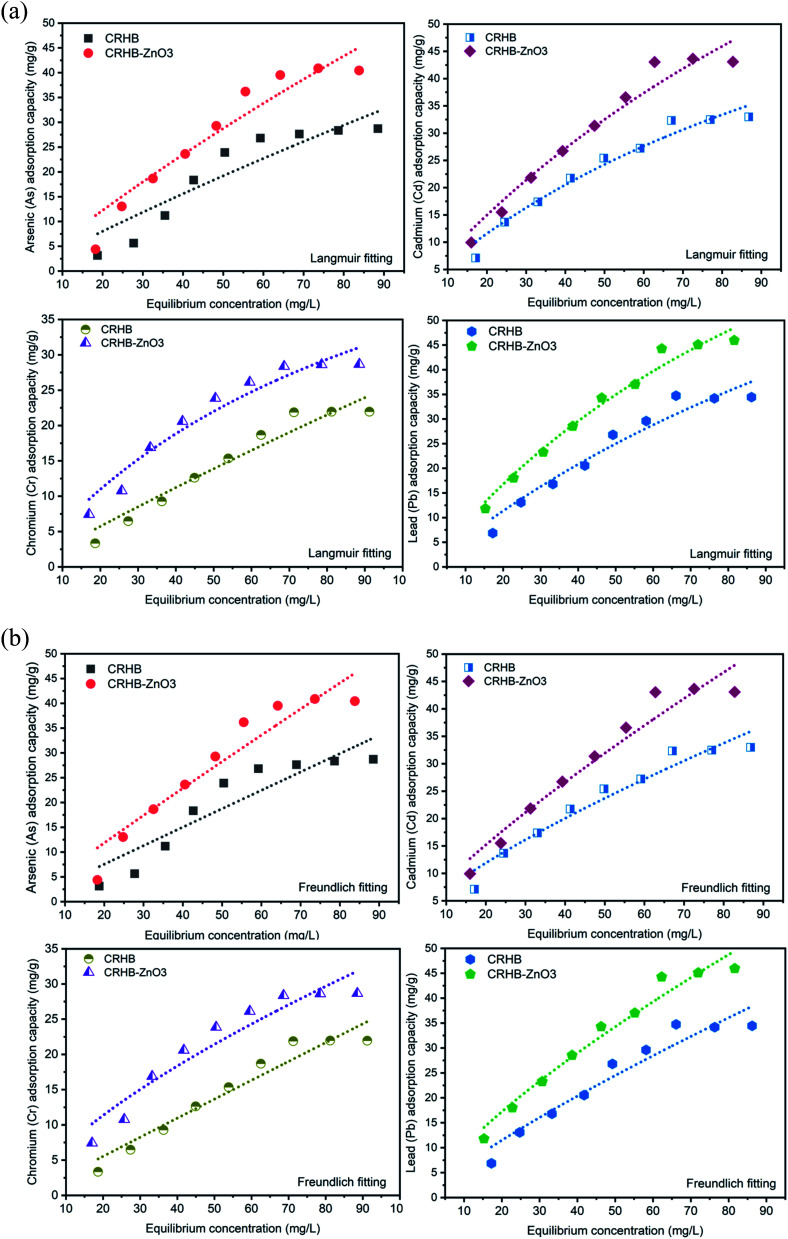
(a) Langmuir isotherm of heavy metals adsorption onto CRHB and CRHB-ZnO3. (b) Freundlich isotherm of heavy metals adsorption onto CRHB and CRHB-ZnO3.

The initial concentration range employed to determine effect of initial heavy metals concentration on adsorption process was from 20–100 mg L^−1^. Solution pH was maintained at the most suitable point of 6 while the adsorption time was 60 min and the applied dose of adsorbents was 0.01 g/25 mL. Records of adsorption capacities of CRHB and CRHB-ZnO3 for each type of heavy metals are illustrated in Fig. 4a.

As similar as the tendency recorded during the contact time experiment, all the four heavy metal adsorption processes were relatively homogeneous in terms of adsorbents' behaviors. In general, the adsorption capacities of CRHB and CRHB-ZnO3 increased significantly as the initial concentration of heavy metals increased from 20 mg L^−1^ to 80 mg L^−1^. At higher initial concentrations of metals, the adsorption capacities were virtually unchanged with an inconsiderable increase in adsorption rate. This also means 80 mg L^−1^ was the parameter that yielded the highest efficiency for the adsorption of heavy metals onto CRH-based adsorbents. To be specific, at initial all heavy metals concentrations of 20–80 mg L^−1^, the As(iii) adsorption capacities of CRHB and CRHB-ZnO3 rose from 3.14–25.34 mg g^−1^ and 7.40–39.52 mg g^−1^, respectively. For Cd(ii), the changes in terms of adsorption capacities from the lowest initial concentration to the peak of Cd(ii) adsorption efficiency onto CRHB and CRHB-ZnO3 were from 7.11–32.33 mg g^−1^ and 9.96–43.05 mg g^−1^, respectively. Towards Pb(ii), the adsorption capacities of CRHB and CRHB-ZnO3, respectively, increased from 6.87 and 11.82 mg g^−1^ to 34.74 and 44.27 mg g^−1^. And finally, the Cr(vi) adsorption capacities of CRHB and CRHB-ZnO3 increased from 3.36–21.89 mg g^−1^ and 7.44–28.37 mg g^−1^, respectively. This tendency occurred was due to the proportionality between the dose of adsorbents and the initial concentration of heavy metals. When the initial concentration of heavy metals increased from 20 to 80 mg L^−1^, the adsorption efficiency of metals ions was elevated due to faster diffusion of the ions onto the biochars' surface resulting in a higher adsorption capacity. Nevertheless, when the initial concentration exceeded 80 mg L^−1^, the adsorption capacity had no further growth as the interaction and linking of metals ions with the adsorbent's surface were limited and formed layers causing ultimately adsorption process was stopped. More specifically, the active adsorption sites were fully occupied and could no longer adsorbed any more ions.^58^ The initial concentration growing meant the adsorption process was attaining the equilibrium state between heavy metals and active sites on the adsorbent, which optimized the speed of attachment between adsorbents and adsorbates in the same period of contact time. However, as the heavy metals surpassed the equilibrium, more competition for active sites occurred and less proportion of heavy metals was adsorbed leading to adsorption degraded.^72^ In other words, although the adsorption capacities might increase with initial concentration but the adsorption rate increased insignificantly and the adsorption efficiency was low. This behavior of adsorption process toward changes in initial concentrations of absorbate was also observed in the studies of Alam *et al.*^42^ and Al-Senani and Al-Fawzan.^73^ In conclusion, 80 mg L^−1^ was the best initial concentration of heavy metals applied for CRH-based adsorbents. The result of this experiment was also optimized when it was used in all the next optimal conditions.

In summary, throughout all the batch experiments, it can be clearly seen that among metals adsorption capacities, the adsorption capacities of CRH-based adsorbents toward Pb were the highest. At optimal condition, the adsorption capacities of Pb(ii) reached 34.47 and 44.27 mg g^−1^ for CRHB and CRHB-ZnO3, respectively. The most second adsorbed metal was Cd(ii) with capacities of CRHB and CRHB-ZnO3 of 32.33 mg g^−1^ 42.05 mg g^−1^, respectively. These two adsorbents also exhibited a good performance in the As(iii) adsorption with the capacities of 26.42 and 39.52 mg g^−1^, respectively, corresponding with CRHB and CRHB-ZnO3. Cr(vi) was the least adsorbed metal with only 21.89 mg g^−1^ for CRHB and 28.37 mg g^−1^ for CRHB-ZnO3 in the same conditions of experiments. In conclusion, the order from the highest adsorption capacity to the lowest for both adsorbents was Pb(ii) > Cd(ii) > As(iii) > Cr(vi) in this study.

The results of experiments assessing the effect of initial heavy metals concentrations on adsorption performed by CRHB and CRHB-ZnO3 were applied to describe adsorption isotherms by Langmuir and Freundlich models. The compatibility of these models with the adsorption process is shown in Fig. 4a (Langmuir model) and Fig. 4b (Freundlich model), while calculations of adsorption isothermal parameters are presented in Table 3.

**Table 3:** Computed isothermal parameters for heavy metals adsorption onto CRHB and CRHB-ZnO3

	As(iii)		Cd(ii)		Pb(ii)		Cr(vi)	
	CRHB	CRHB-ZnO3	CRHB	CRHB-ZnO3	CRHB	CRHB-ZnO3	CRHB	CRHB-ZnO3
**Langmuir isotherms**								
*q*_e,cal_ (mg g^−1^)	25.78	35.89	29.79	38.65	31.02	40.75	19.35	26.90
*K*_L_ (L mg^−1^)	0.002	0.002	0.0075	0.006	0.005	0.008	0.001	0.009
*R* ^2^	0.863	0.922	0.967	0.963	0.971	0.950	0.945	0.948

**Freundlich isotherms**								
*K*_F_ (mg g^−1^) (mg L^−1^)^*n*^	0.391	0.695	1.256	1.351	0.974	1.819	0.291	1.425
*n*_F_ (g mg^−1^ min^−1^)	1.010	1.056	1.331	1.237	1.213	1.333	1.017	1.443
*R* ^2^	0.858	0.915	0.950	0.951	0.936	0.969	0.945	0.921

In general, the adsorption capacity for each metal was still in the order of Pb(ii) > Cd(ii) > As(iii) > Cr(vi). Nevertheless, the correlation coefficients were 0.863–0.971 and 0.858–0.970 for Langmuir and Freundlich models, respectively. For Langmuir isothermal model, the *K*_L_ values were between 0.0012–0.0075 and 0.0023–0.0099 for the adsorption on to CRHB and CRHB-ZnO3, respectively. As these values were within 0–1, the adsorption was well described by the Langmuir model. For Freundlich isotherms, the *n* values were within 1.012–1.331 (for CRHB) and 1.056–1.443 (for CRHB-ZnO3). The adsorption of heavy metals onto CRHB-ZnO3 resulted in the *n* values of Freundlich greater than 1 proved the adsorption process was controlled by chemisorption mechanism. While for CRHB, the *n* values were smaller than 1 (except for Cd(ii)), suggesting physical adsorption mechanism with weak interactions applied on the adsorption process. The calculated adsorption capacities of CRHB and CRHB-ZnO3 from both Langmuir and Freundlich models were well fitted to the experimental data obtained from this study (Table 4). However, the results of adsorption capacities onto both CRHB and CRHB-ZnO3 obtained from fitting Langmuir model (Table 3) were closer to the actual data obtained from experiments (Table 4) compared with Freundlich model. This suggests the adsorption of As(iii), Cd(ii), Pb(ii), and Cr(vi) was monolayer and homogenous on the active sites of CRHB-based adsorbents.^56,74^ Langmuir isothermal model was also reported to better describe the adsorption of heavy metals onto coconut husk^41^ and rice husk adsorbents.^40^

**Table 4:** The maximum adsorption capacities of CRHB and CRHB-ZnO3 obtained from data study on effect of initial heavy metals concentrations on metals adsorption

	Adsorption capacity (mg g^−1^)				Total (mg g^−1^) (As(iii), Cd(ii), Pb(ii) and Cr(vi))
	As(iii)	Cd(ii)	Pb(ii)	Cr(vi)	
CRHB	26.42	32.33	34.47	21.89	115.11
CRHB-ZnO3	39.52	42.05	44.27	28.37	154.21

### 3.5. Characteristic of adsorbents and adsorption mechanisms

**Fig. 5:**
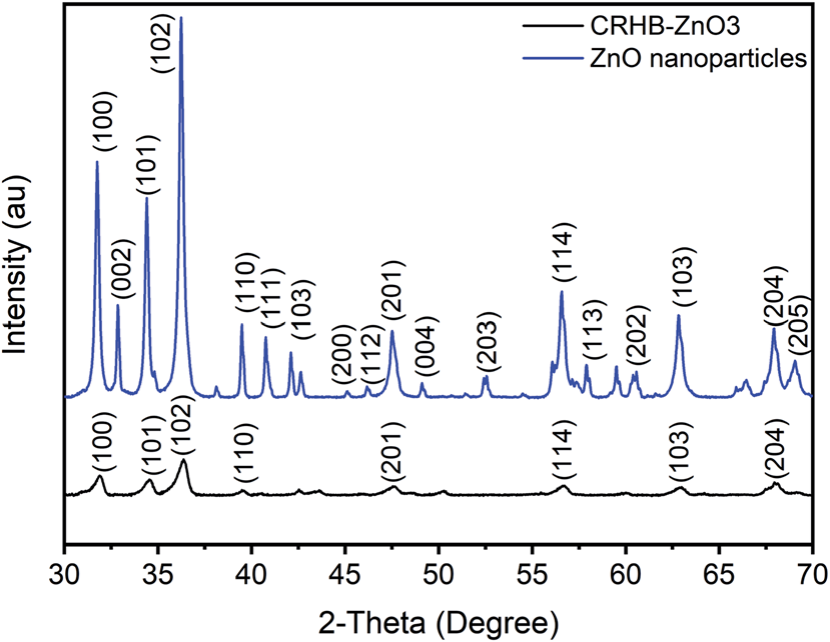
Graph of XRD ZnO nanoparticles and CRHB-ZnO3.

**Fig. 6:**
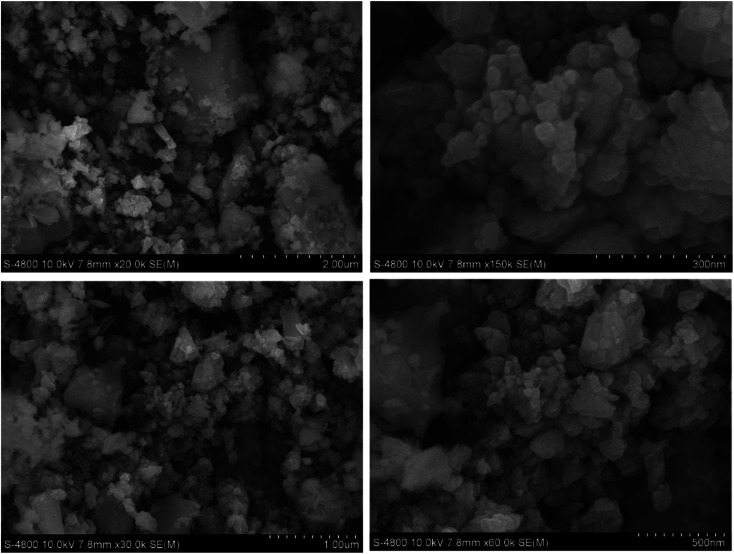
Field emission scanning electron microscopy (FESEM) images of ZnO nanoparticles.

**Fig. 7:**
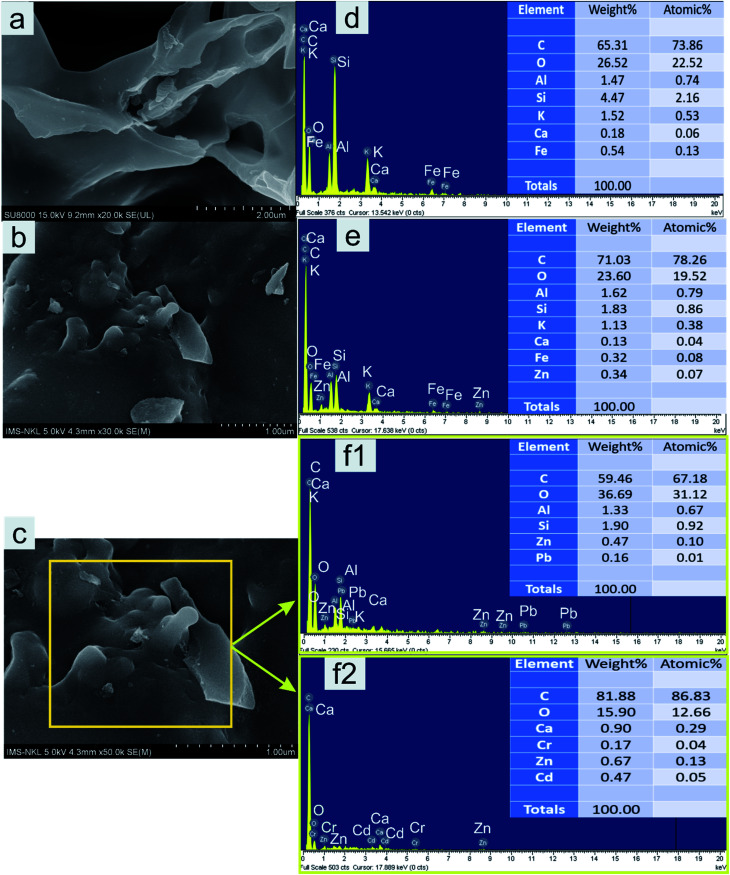
SEM images and EDS profiles of CRHB (a and d) and CRHB-ZnO3 (b and e) before and after adsorption (c, f1 and f2).

Theoretically, the modification of cassava root husk-derived biochar with ZnO-NPs was desired providing a more heterogeneous structure compared with the pristine adsorbent. That was due to combination of two distinct components in one which should result in a variable composition. Secondly, the lighter and smaller nanoparticles should increase the surface area of the modified adsorbent. The crystallinity, phase, and purity of ZnO nanoparticles and CRHB-ZnO3 were characterized using the powder XRD analysis data (Fig. 5). The presence of ZnO particles is shown at major refection peaks of 31.76°, 32.93°, 34.42°, 36.22°, 39.53°, 40.77°, 42.08°, 45.09°, 46.17°, 47.51°, 49.09°, 52.54°, 56.56°,57.87°, 60.57°, 62.79°, 67.9° and 69.05°, corresponding to (100), (002), (101), (102), (110), (111), (103), (200), (112), (201), (004), (203), (114), (113), (204) and (205) for XRD of ZnO nanoparticles (JCPDS card no. 01-075-0576) and eight major peaks of 31.87°, 34.65°, 36.31°, 39.52°, 47.51°, 56.86°, 62.78° and 67.91°, identifying to (100), (101), (102), (110), (201), (114), (103) and (204) for CRHB-ZnO3 (JCPDS card no. 01-075-0576). These results indicate that the ZnO nanoparticles were successfully loaded on the CRHB. Furthermore, FESEM images of ZnO nanoparticles are presented in Fig. 6. The porous and rough overall shape with much small particles can be easily observed from the morphology of the particles. In fact, this was well demonstrated through the SEM data of both CRHB and CRHB-ZnO3 before (Fig. 7a and b), respectively, and after (Fig. 7c) adsorption. Both CRHB and CRHB-ZnO3 possessed surface structures that were porous and rough.

Based on the data provided by Table 5, it is clear that the BET surface area of CRHB was marginally elevated after being modified with ZnO-NPs. Particularly, CRHB only had a surface area of 1.9056 m^2^ g^−1^. While the BET surface area of CRHB-ZnO3 increased by approximately 46.75% (2.7964 m^2^ g^−1^). Moreover, CRHB-ZnO3 was dominant to CRHB in terms of porosity when its pore volume measured was 0.904 cm^3^ g^−1^ compared with only 0.00108 cm^3^ g^−1^ of CRHB. This leads to a certain outcome that with a larger surface area and more porous structure, CRHB-ZnO3 was completely capable of outperforming CRHB in adsorption with more spaces for heavy metals to be attached on it. Although, as desired that ZnO-NPs-modification would enhance textural characteristics of modified biochar but it is clear from data in Table 5 that both applied biochars were classified as non-porous materials which led to contribution of physical adsorption mechanism (*i.e.* pore filling) was negligible. The results were suitable with adsorption isothermal analysis discussed in detail in next section.

**Table 5:** Physical properties of CRHB and CRHB-ZnO3

Biochar	BET surface area (m^2^ g^−1^)	Pore volume (cm^3^ g^−1^)	pH_PZC_
CRHB	1.9056	0.00108	8.25
CRHB-ZnO3	2.7964	0.9040	6.94

**Fig. 8:**
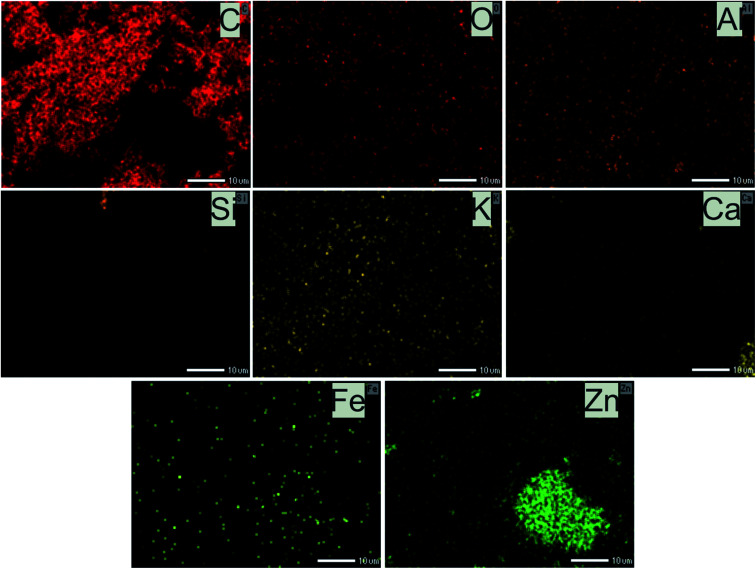
Mapping data of CRHB-ZnO3.

The physical–chemical characteristics of biochars were further confirmed by the EDS data (Fig. 7) which illustrated the composition of both adsorbents. What stands out from data in Fig. 7 is that main constituents in CRHB's weight were accounted by the components of organic compounds like carbon (65.31%) and oxygen (26.52%). Other components, which included Fe, Al, Si, Ca, and K, only occupied 8.18% of the total weight and 3.62% in terms of atoms (Fig. 7d). The compositions of CRHB-ZnO3 were relatively similar to those of CRHB. However, there was the presence of 0.34% Zn (0.07% of the atoms) that was different from those of CRHB (Fig. 7e). These results were also in agreement with the mapping data of CRHB-ZnO3 in Fig. 8. These images confirmed the presence of C, O, Al, Si, K, Ca, Fe and Zn elements in biochars' constituent. This index pointed out the success of loading ZnO-NPs onto CRHB and a slight inconsiderable decrease of other components in pristine biochar. In addition, Fig. 7f1 and f2 provide the EDS analysis of CRHB-ZnO3 at two different active sites after adsorption. The obtained data proved that the distribution of components varied through these active sites. And when the Pb element was detected in Fig. 7f1, there were presence of Cr(vi) and Cd elements in Fig. 7f2. This also indicated that Cr(vi), Pb(ii), and Cd(ii) were successfully adsorbed on the surface of CRHB-ZnO3.

**Fig. 9:**
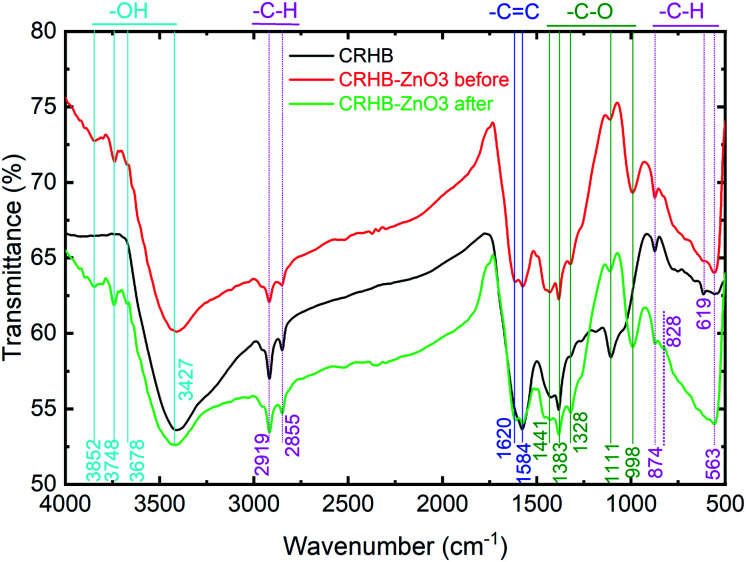
FTIR of CRHB and CRHB-ZnO3 before and after the adsorption of heavy metals.

Fig. 9 shows the FTIR analysis data of both CRHB and CRHB-ZnO3 before and after adsorption which consisted of the available surface functional groups data on the adsorbents. Remarkably, the hydroxide groups (–OH), which are presented at peaks of 3852, 3748, and 3678 cm^−1^ were clearly detected on CRHB-ZnO3 before and after adsorption but not on CRHB. However, there was a slight drop in peak area of –OH after adsorption which showed the participation of oxygen-containing surface groups into metals adsorption by biochars. The similar result was obtained for the peak of 828 cm^−1^, representing –CH groups. On the other hand, CRHB had a peak at 619 cm^−1^ (–CH), which was not detected on CRHB-ZnO3 before and after adsorption which showed that ZnO-NPs-treated biochar reduced aromatic ring structure of pristine biochar. Besides, all examined samples shared common peaks corresponding –CH groups (3427 cm^−1^), –CH (2855, 2919, 874, and 563 cm^−1^), –C=C (1620 and 1584 cm^−1^) which was characteristic of aromatic ring organic compounds. Especially, the abundant presence of –CO– at 1441, 1383, 1328, 1111, and 998 cm^−1^ in modified biochar before adsorption which was not detected in pristine biochar, suggesting that ZnO-NPs-modified biochar was enriched the oxygen-containing surface groups which played a major role in adsorption mechanisms of metal cations by biochars.

The data of pH_PZC_ values of the two adsorbents are also indicated on Table 5 which further supported for deeply discussion about metals adsorption behaviors onto biochars. The pH_PZC_ of CRHB was 8.25 and that of CRHB-ZnO3 was 6.94. Therefore, the modification of CRHB with ZnO-NPs dropped the pH_PZC_ value as the Zn(ii) ions, a bisexual metal, were formed on the surface of CRHB during the loading process. The better adsorption capability of CRHB-ZnO3 compared with CRHB also indicated that ZnO-NPs played an important role in the adsorption of heavy metals mainly contribution of oxygen-containing surface groups enriched on ZnO-NPs-modified biochar but not textural properties of adsorbent. Besides, the ZnO nanoparticles loaded on CRHB also caused the aromatization of the carbon skeleton which resulted in the enhancement of adsorption ability of modified biochar.^75^ That was reason why the adsorption capacities of CRHB-ZnO3 towards As(iii), Cd(ii), Pb(ii), and Cr(iv) were significantly higher compared with CRHB.

The EDX analysis (Fig. 7f1 and f2) and mapping data (Fig. 8) show that after adsorption, the presence of Pb(ii), Cd(ii), and Cr(vi) was observed, proving that precipitation occurred on the surface of CRHB-ZnO3. That was because the linkage between the metal ions and the –OH groups or the –CO groups on the adsorbent's surface. Furthermore, Ca and Al elements were found as a component of the biochars before adsorption (Fig. 7f1) but they were virtually undetected after adsorption (Fig. 7f2). This suggests there was an ion exchange process occurring between Ca and Al with heavy metal ions within the solution. In addition, K element was not found in EDS analysis data of CRHB-ZnO3 after adsorption, which was possibly because K^+^ ions were also participated in ion exchange mechanism. Clearly, this was the evident for the adsorption mechanism stated in the adsorption kinetics discussions on which chemisorption was based on interactions among oppositely charged components of the process. Therefore, the adsorption of heavy metals onto CRHB and CRHB-ZnO3 occurred based on major mechanisms of ion exchange and surface precipitation.

In addition, the role of ZnO-NPs further asserted the promotion of the uptake of heavy metals onto the adsorbent. Particularly, as the ZnO-NPs modification biochar was widened in terms of surface area and porosity, the rate of particle diffusion onto the surface increased and adsorption occurred as a result. The study of Gu *et al.*^76^ regarding selective heavy metal adsorption using ZnO-NPs referred to this feature as a mechanism of adsorption. The interaction of ZnO-NPs with the composition of the adsorbent before and after adsorption in this study was also a proof of stabilization which further confirmed highly application of this material for removing heavy metals from contaminated water.^77^ The contribution of ZnO-NPs towards adsorption mechanisms in this study was also agreed with the study of Nalwa *et al.*^78^

Compared to other adsorbent materials used for removal of heavy metals, the CRHB and CRHB-ZnO3 possessed the BET surface area that is lower than the area of iron-coated Australian zeolite Fe_3_O_4_@SiO_2_–EDTA nano composite, but higher than the area of magnetic modified biochar derived from raw corncob (Table 6). The porosity of CRHB and CRHB-ZnO3 appeared slightly higher than other materials (*Padina gymnospora*, iron-coated Australian zeolite). Functional groups available on CRHB-based adsorbents are mainly –OH, C–H, C=C, C–O. These are similar to the functional groups of other materials. Nevertheless, the quantity is lower. *Padina gymnospora* has some extra groups such as N–H and S–H. Aminopyrazole modified graphene oxide possessed C–OH and N–H. Magnetic modified corncob biochar shared the abundance of C≡C. On the other hand, iron-coated Australian zeolite only had O-T-O stretching vibration. For Fe_3_O_4_@SiO_2_–EDTA nano composite, Fe–O bond, –OH, Si–O–C, Si–O–Si, Si–OH, C=O, –COOH groups were dominant. The adsorption capacity toward heavy metals of CRHB and CRHB-ZnO3 in this study was higher than those of magnetic modified biochar and iron-coated Australian zeolite. However, its efficiency was lower than those of *Padina gymnospora* and Fe_3_O_4_@SiO_2_–EDTA nano composite. In general, when it came to adsorbing heavy metals in combination, the results were high. Both CRHB and CRHB-ZnO3 were completely capable of well performing in solutions that simultaneously contained As(iii), Cd(ii), Pb(ii), and Cr(vi). Particularly, the total adsorption capacities of the two adsorbents were, respectively, 82.98 and 150.83 mg g^−1^. Therefore, the potentials of applying this material in treating heavy metals-contaminated water sources are high.

**Table 6:** Comparison of heavy metal adsorption capacities using CRHB-ZnO3 with adsorption capacities of reported other adsorbents

Adsorbent	Characteristics	Heavy metal ions	*q*_max_ (mg g^−1^)	Ref.
*Padina gymnospora*	SEM: smooth, micro and macro pores; functional groups: –OH, N–H, –CH, S–H, C=C, C–O	Cd(ii), Cr(iii)	96.46, 31.52	62
3-Aminopyrazole modified graphene oxide	FE-SEM: sp^2^-hybridized carbon atoms, crumpled edge; functional groups: C=C, C=O, C–O, C–OH, N–H, –CH	Cd(ii), Hg(ii) and As(iii) ions	285.714, 227.273, and 131.579	61
Magnetic modified biochar derived from raw corncob	BET: 1.49 m^2^ g^−1^, pore volume: 0.0031 cm^3^ g^−1^, SEM: porous and rougher, functional groups: –OH, C=C, C≡C, C–O, RXD: C-graphite, Fe_3_O_4_	Cr(vi) iron	25.94	58
Iron-coated Australian zeolite	BET: 7.51 g m^−2^, functional groups: O–T–O stretching vibration	Pb(ii), Cu(ii), Cd(ii), Cr(vi), Zn(ii) ions	5.0–11.2 (single metal), 3.7–7.6 (mixed metals)	79
Fe_3_O_4_@SiO_2_–EDTA nano composite	BET: 24.1 m^2^ g^−1^, pore size: 8.3 nm, pore volume: 2.2 × 10^−3^ cm^3^ g^−1^, functional groups: Fe–O bond, –OH, Si–O–C, Si–O–Si, Si–OH, C=O, –COOH	Cu(ii), Cd(ii) irons	79.4, 73.5	80
CRHB	BET: 1.91 m^2^ g^−1^, pore volume: 0.00108 cm^3^ g^−1^, SEM: porous and rough, functional groups: –OH, C–H, C=C	As(iii), Cd(ii), Pb(ii), and Cr(vi) ions	28.34, 26.42, 32.33 and 21.89 (mixed metals)	This study
CRHB-ZnO3	BET: 2.79 m^2^ g^−1^, pore volume: 0.9040 cm^3^ g^−1^, SEM: porous and rough, functional groups: –OH, C–H, C=C, C–O	As(iii), Cd(ii), Pb(ii), and Cr(vi) ions	40.89, 39.52, 42.05 and 28.37 (mixed metals)	This study

## 4. Conclusions

Through all the adsorption experiments, it was obvious that the cassava root husk-derived biochar possessed good qualities to act as a high effective adsorbent for the adsorption of trivalent arsenic, cadmium, lead, and hexavalent chromium from water. The potential of this fully promising adsorbent can even be extended when combining with ZnO nanoparticles. The suitable conditions for As(iii), Cd(ii), Pb(ii), and Cr(vi) adsorption by CRHB and CRHB-ZnO3 in aqueous solutions were ZnO impregnation ratio of 3% (w/w), solution pH of 6, contact time of 60 min, and heavy metals initial concentration of 80 mg L^−1^. With such conditions, both employed adsorbents, including CRHB and CRHB-ZnO3 exhibited the adsorption preference in an order of Pb(ii) > Cd(ii) > As(iii) > Cr(vi). The maximum adsorption capacities of CRHB and CRHB-ZnO3 could attain 28.34 and 40.89 mg g^−1^, respectively, for the adsorption of arsenic (As(iii)); 26.42 and 39.52 mg g^−1^ for the adsorption of cadmium (Cd(ii)); 32.33 and 42.05 mg g^−1^ for lead (Pb(ii)) adsorption; and 21.89 and 28.37 mg g^−1^ for chromium (Cr(vi)) adsorption. In terms of isotherms, the Langmuir model was the most suitable to describe the adsorption behaviors of heavy metals onto biochars. The adsorption experimental data of heavy metals onto biochars were well fit with both pseudo-first-order and pseudo-second-order models. The mechanisms of heavy metals adsorption onto biochars were chemisorption occurring homogeneously in terms of energy on monolayers by ion exchange, surface precipitation and pore filling. This study was successfully developed a low-cost, high-effective and eco-friendly adsorption material with combination between agricultural by-product derived-biochar and ZnO nanoparticles. However, the study was limited at the lab-scale with tests on the simulated wastewater, thus it is necessary that the study should be scaled-up with application in practical for removal of heavy metals from real wastewater in the future.

## Conflicts of interest

The authors declare that they have no conflict of interest.

## Supplementary Material
